# Assessing Global DNA Methylation Changes Associated with Plasticity in Seven Highly Inbred Lines of Snapdragon Plants (*Antirrhinum majus*)

**DOI:** 10.3390/genes10040256

**Published:** 2019-03-28

**Authors:** Delphine Gourcilleau, Mathilde Mousset, Mathieu Latutrie, Sara Marin, Alain Delaunay, Stéphane Maury, Benoît Pujol

**Affiliations:** 1 Laboratoire Évolution & Diversité Biologique (EDB, UMR 5174), Université Fédérale de Toulouse Midi-Pyrénées, CNRS, IRD, UPS. 118 route de Narbonne, Bat 4R1, CEDEX 9, 31062 Toulouse, France; mathilde.mousset@gmail.com (M.M.); mathieu.latutrie@univ-tlse3.fr (M.L.); sara.marin@univ-tlse3.fr (S.M.); 2 PSL Université Paris: EPHE-UPVD-CNRS, USR 3278 CRIOBE, Université de Perpignan, 52 Avenue Paul Alduy, CEDEX 9, 66860 Perpignan, France; 3 Laboratoire de Biologie des Ligneux et des Grandes Cultures (LBLGC, EA 1207 USC 1328 INRA), Université Orléans, 45067 Orléans, France; alain.delaunay@univ-orleans.fr (A.D.); stephane.maury@univ-orleans.fr (S.M.)

**Keywords:** phenotypic plasticity, DNA methylation, shade avoidance, stem elongation, snapdragon

## Abstract

Genetic and epigenetic variations are commonly known to underlie phenotypic plastic responses to environmental cues. However, the role of epigenetic variation in plastic responses harboring ecological significance in nature remains to be assessed. The shade avoidance response (SAR) of plants is one of the most prevalent examples of phenotypic plasticity. It is a phenotypic syndrome including stem elongation and multiple other traits. Its ecological significance is widely acknowledged, and it can be adaptive in the presence of competition for light. Underlying genes and pathways were identified, but evidence for its epigenetic basis remains scarce. We used a proven and accessible approach at the population level and compared global DNA methylation between plants exposed to regular light and three different magnitudes of shade in seven highly inbred lines of snapdragon plants (*Antirrhinum majus*) grown in a greenhouse. Our results brought evidence of a strong SAR syndrome for which magnitude did not vary between lines. They also brought evidence that its magnitude was not associated with the global DNA methylation percentage for five of the six traits under study. The magnitude of stem elongation was significantly associated with global DNA demethylation. We discuss the limits of this approach and why caution must be taken with such results. In-depth approaches at the DNA sequence level will be necessary to better understand the molecular basis of the SAR syndrome.

## 1. Introduction

In nature, organisms are often confronted with heterogeneous environments. Phenotypic plasticity is the capacity for an organism to produce several phenotypes in response to changes of its biotic and abiotic environments [1,2,3]. When adaptive, plasticity allows organisms to cope rapidly with new environmental constraints [4,5]. Genetic and ecological mechanisms that underlie the diversity of plastic responses to environmental cues are widely documented [6]. One possibly involved molecular mechanism that resurfaced in the scientific literature is epigenetics [7,8,9,10,11]. Epigenetic variation is commonly used today to refer to molecular mechanisms influencing gene expression that affect the phenotype without any change of DNA sequence [12], although it historically encompassed a broad range of developmental and phenotypic variation [13]. For example, chromatin dynamics—changes in the organization and structure of the chromatin—can be caused by DNA methylation or histone post-translational modifications, and they modify gene expression and transposable element (TE) mobility [14,15,16,17,18]. There is growing evidence for epigenetic variation associated with trait variation and phenotypic plasticity [8,9,10,11,19,20]. Epigenetic variation plays a role in various processes resulting in modifications of the phenotype, e.g., development and specific responses to biotic or abiotic environmental stressors (for reviews, see Reference [21] or [22]). Several examples of phenotypic plasticity associated with epigenetic changes can be found in the literature: response to abiotic stresses such as heat [23] or cold [24] and to biotic stress such as pathogens [25]. It remains nevertheless necessary to direct research efforts toward documenting the diversity of these associations in order to obtain a more global picture of epigenetic mechanisms and to assess whether their ecological significance can be generalized.

The most documented case of phenotypic plasticity in plants is their shade avoidance response (SAR) [26]. It is a common syndrome that includes multiple trait responses such as changes in internode length (stem elongation), apical dominance (reduced branching), and photosynthetic efficiency (increased specific leaf area; SLA) [26,27]. Most plants perceive changes in the quantity, quality, and direction of light (for a review, see Reference [28] or [29]). The physiology of the SAR is well documented [30]. There is evidence that phytochromes A/B/C and phytohormones (e.g., auxin, ethylene, and gibberellins) are involved in this mechanism [31,32,33,34]. Its ecological and evolutionary significances are widely acknowledged [26]. It can be adaptive in the presence of competition for light. For example, stem elongation in response to shade can allow plants to reach sunlight and pollinators in a densely populated habitat, thereby increasing their fitness [35,36,37]. It might play an important role in the adaptive potential of plant populations to environmental changes because contemporary changes in vegetation cover are observed worldwide, as a result of fragmentation [38] and land-use changes [39]. SAR is also known to have evolved in response to selection (e.g., during domestication [40]). Collectively, these aspects call for assessing whether epigenetic variation is associated with the widely distributed and documented SAR, particularly to improve our knowledge on the ecological significance of epigenetic variation. 

SAR is a syndrome involving complex traits governed by multiple pathways associated with multiple genes, e.g., *PIF*, *HAT*, *ATHB2*, or *YUC* [30]. For example, these genes were identified in *Arabidopsis*, maize, tomato, and tobacco [26,30,32,34,41], and so were their interactions and regulation [30]. There is also evidence for the genetic variation of SAR and its potential to evolve in response to selection [37,42,43,44]. Genetic mechanisms underlying the SAR are widely described, while next to little is known in terms of epigenetic variation. There are, to our knowledge, only two studies documenting the epigenetic basis of SAR [45,46]. Tatra et al. (2000) submitted two clonally propagated genotypes of *Stellaria longipes* to two light treatments (3.7 and 0.7 red/far-red ratio). They followed stem elongation and methylated cytosine content using high-performance liquid chromatography (HPLC) and found a correlation between the stem elongation response and levels of demethylation. They also submitted plants to 5-azacytidine treatment (a demethylating agent) to confirm the implication of DNA methylation in stem elongation and possibly in the SAR [46]. Peng et al. (2018) showed that acetylation of H3/H4 and H3K4me3/H3K36me3 promoted the expression of shade responsive genes in the Col-0 genotype of *Arabidopsis thaliana*. Knowledge about the epigenetic basis of SAR, therefore, remains incomplete. Whether it can be generalized to other plant species remains to be addressed. 

Here, we present results from our investigation of the epigenetic basis of the shade avoidance response in seven highly inbred lines of *Antirrhinum majus*. Extensive work on developmental genomics was done on *A. majus* [47], which was shown to react in terms of growth and development to light quality and intensity [48,49]. Its natural populations are highly genetically diverse [50,51,52] and geographically distributed across a large range of environmental conditions, particularly in terms of vegetation cover [53]. It is, therefore, a good system to study the genetic and epigenetic variation underlying the SAR. We submitted each *A. majus* line to shade treatments, which allowed us to study a fixed genetic background between individuals within lines, thereby excluding or extremely reducing the effect of genetic variation. Although epigenomics methods such as whole-genome bisulfite sequencing (WGBS) can provide detailed information at the scale of the cytosines over the whole genome [54], these approaches require a complete reference genome. *A. majus* reference genome was published in 2019, after we conducted this study [55]. Other approaches that do not require a reference genome are also available (e.g., epigenome bisulfite sequencing (epiGBS), bisulfite converted restriction site associated dna sequencing (bsRADseq), Epi RADseq, and methylated DNA immunoprecipitation (meDIP) [56,57,58,59]). However, the use of these methods is still restricted to small sample sizes that limit the epigenomic characterization of multiple populations and, therefore, the investigation of ecologically relevant variation at the population level. Epigenetic variation different from methylation changes might also be worth investigating (e.g., histone modifications using a ChipSeq approach or non-coding RNAs using an RNAseq approach). Here, we assessed the global DNA methylation (%mC). Although this approach does not provide epigenetic characterization at the detailed level of genes and genomic regions, it is a widely used and proven epigenetic indicator in plants accessible at the population level [60,61,62,63,64,65]. We chose to estimate the %mC in the shoot apical meristems using the most approved HPLC technique [66]. Shoot apices were chosen because it is the place where new tissues start their differentiated growth and development. This is also where plants perceive external signals that drive phenotypic responses linked to growth or development. 

## 2. Material and Methods

### 2.1. Plant Material

*Antirrhinum majus* L. (snapdragon) is a hermaphroditic, self-incompatible, short-lived perennial plant from the *Plantaginaceae* family, which produces annual inflorescences with zygomorphic flowers. In this study, we used seven highly inbred lines of *A. majus*. These lines were generated by producing successive generations of self-fertilization. Their inbred genetic background was, therefore, mostly fixed at the homozygous state and was used as genetic stock to generate particular variants through inter-line crosses. Six lines were obtained from the John Innes Center (Norwich Research Park, Dr Lucy Copsey), namely Ji2, Ji7, Ji75, Ji98, Ji522, and Si50. The seventh line was provided by the Technical University of Cartagena (Instituto de Biotecnología Vegetal, Pr Marcos Egea Gutiérrez-Cortines), namely E165. 

### 2.2. Experimental Design

Plant cultivation was conducted in a greenhouse at the Center for National Scientific Research (CNRS) experimental station in Moulis (France). A total of 20 plants per line (*N* = 140) grew under controlled environmental conditions with a large input of natural sunlight that was supplemented by artificial lighting (High-Pressure Sodium 400 lights, Hortilux Shreder, Monster, The Netherlands) when the photoperiod was <16 h and/or when the light intensity was <400 watt/m^2^. Temperature was maintained below 30 °C by high-pressure water cooling and above 15 °C by central heating. Seeds were sown on 8 March 2017 in a mixture compost (50% BP2 Kompact 294, 50% TS3 Argile 404; Klasmann, Bourgoin Jallieu, France). Soon after all seeds germinated, seedlings were transplanted in individual 9 × 9 cm pots containing the same mixture compost (28 March 2017). Plants were watered approximately twice each week. Shade treatments were applied on 6 April 2017 when all seedlings had developed one or two internodes. We subjected the seven lines to four treatments (regular light, 15% shade, 45% shade, 70% shade) using three different types of shading nets. For logistical reasons, shade treatments were applied under controlled conditions as blocks that were not replicated. Five plants per line were subjected to each treatment. Plants were randomly distributed within treatments. We estimated the degree of shade produced by nets using spectrophotometer acquisitions (Figure S1, Supplementary Materials).

### 2.3. Phenotypic Measurements

We estimated the shade avoidance response 40 days after applying shade treatments (16 May 2017). Phenotypic measurements included plant height in cm, number of branches, number of floral buds, number of internodes, and stem diameter in mm. Internode length was calculated as the average stem length in cm per internode (plant height/number of internodes). Five fully developed leaves were collected and directly scanned on a regular scanner (CanoScan LiDE 220, Canon, Tokyo, Japan) after harvest to avoid any water loss (see Reference [67] for methods). They were then dried in an oven (three days at 45 °C) and weighted. The area of leaves was measured using the ImageJ software [68], and the SLA was calculated as the sum of the areas of the five leaves (m^2^)/sum of dry masses of the five leaves (kg).

### 2.4. Global DNA Methylation (%mC)

At the end of the cultivation experiment, the shoot apex of each plant was harvested, frozen in liquid nitrogen, and conserved at −80 °C until epigenetic analyses. Frozen shoot apices were ground to powder using Tissue Lyser II (Qiagen, Hilden, Germany), which grinds sample tissues by high-speed shaking of plastic tubes containing samples and stainless-steel beads. Total DNA was extracted from plant tissue using the Biosprint 15 DNA Plant kit (Qiagen, Hilden, Germany), which is an automated method. DNA was enzymatically hydrolyzed into nucleosides and was analyzed by high-performance liquid chromatography (HPLC) following a previously published protocol [69,70] with the minor adaptation and control procedure of Zhu et al. (2013) [71]. Five to ten micrograms of DNA in 50 µL of H_2_O was used for these HPLC analyses. Hydrolysis of purified DNA into nucleosides was performed successively using DNase I (700 U, Roche Diagnostics, Meylan, France), phosphodiesterase I (0.05 U, SerLabo Technologies, Entraigues sur la Sorgue, France), and alkaline phosphatase type III (0.5 U, Sigma-Aldrich, Saint-Quentin Fallavier, France). The global percentage of methylation was determined by HPLC [72] using a GeminiTM column (150 × 9 × 4.6 mm, 5 µm, Phenomenex, Le Pecq, France) with an isocratic mobile phase composed of 0.5% methanol (*v*/*v*) and 5 mM acetic acid in water according to the methods of Gourcilleau et al. (2010). Controls for this procedure included co-migration with commercial standards (Sigma-Aldrich), confirmation by enzyme restriction analysis [69], and tests for RNA contamination using HPLC detection of ribonucleosides. The methylcytosine percentages (%mC) were calculated as follows:

%mC = (mC/(C + mC)) × 100,

where C represents 2-deoxycytidine content and mC represents 5-methyl-2-deoxycytidine content. For each sampled apex, we used an average estimate of the %mC based on three independent values of %mC calculated on the basis of three independent HPLC analyses.

### 2.5. Statistical Analysis

All statistical analyses were performed in R version 3.4.3 [73] using the lme4 package [74]. As a preliminary step in the analysis of our data, we evaluated for every trait (plant height, internode length, stem diameter, number of flowers, number of branches, and SLA) whether the relationship between shade treatments (as a continuous effect) and trait values was linear or quadratic. Significance levels of exclusion probabilities (*p*-values), the amount of variation explained by the relationship (*R*^2^), and an indicator of the goodness of fit of the models (AIC) were used to estimate which models had the best fit. We then used this information to define the phenotypic reaction norm of traits.

As a first step, we explored the global effect of single factors on the data using unifactorial generalized linear models (GLM) for the sake of clarity and comparison with later results, with shade treatments considered as a continuous variable. We estimated the effects of (1) shade treatments (as a continuous effect) on phenotypic trait values, (2) shade treatments on global DNA methylation (%mC), and (3) the relationship between phenotypic trait values and %mC. We performed multiple comparison tests between treatments using Kruskal–Wallis tests when we found a significant effect of shade treatments on a trait. As a second step, we used generalized linear mixed models (GLMM) to include the random effect of lines in order to estimate genetic variation amongst lines. We tested for the effect of shade treatments (continuous fixed effect) on trait values, for the effect of lines (random effect) on the trait response to shade (line effect on the slope), and for the effect of lines (random effect) on trait values measured in full light conditions (i.e., line effect on the intercept of the phenotypic response to shade). We also tested for the effect of shade treatments (continuous fixed effect) on %mC, for the effect of lines (random effect) on the changes of %mC in response to shade (i.e., line effect on the slope), and for the effect of lines (random effect) on the %mC measured in full light conditions (i.e., effect of line on the intercept of the %mC changes in response to shade). We then tested for the effect of %mC (continuous fixed effect) on trait values, for the effect of lines (random effect) on the relationship between phenotypic trait values and %mC (line effect on the slope), and for the effect of lines (random effect) on trait values measured at baseline levels of %mC (intercept of the relationship between %mC and trait values). Finally, in order to take into account that only plants reacting to shade are expected to undergo changes in %mC, we tested for a relationship between the magnitude of the trait responses to shade (using the slope of trait reaction norms) and the magnitude of %mC changes in response to shade (using the slope of the %mC reaction norm). This linear relationship was evaluated using a generalized linear model.

## 3. Results

### 3.1. Phenotypic Response to Shade

**Figure 1:**
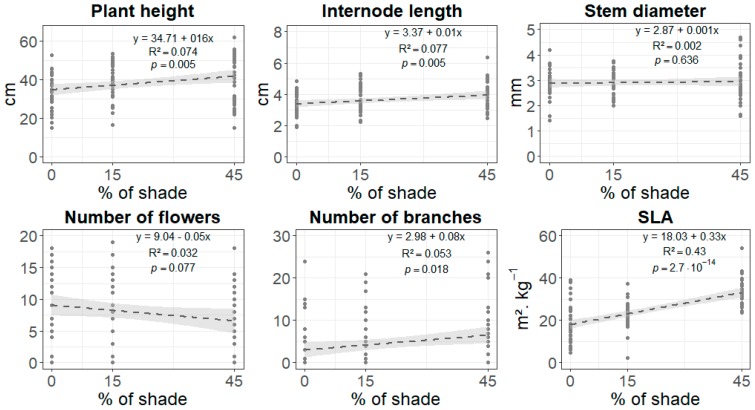
Shade avoidance response in *Antirrhinum majus* highly inbred lines. Linear relationships established by generalized linear models (GLMs) between phenotypic traits and shade. Phenotypic traits included plant height in cm, internode length in cm, stem diameter in mm, number of flowers, number of branches, and specific leaf area (SLA) in m^2^∙kg^−1^. Shade treatments included 0%, 15%, and 45%. Equations, probabilities of significance (*p*), and coefficients of determination (*R*^2^) are given for each relationship. *N* = 105 (five plants × three shade treatments × seven lines). The dotted line represents the regression line surrounded by its 95% confidence interval represented by the gray area.

Our preliminary step in the analysis of the phenotypic response to shade revealed that shade had a significant effect on all the phenotypic traits that we measured: plant height, internode length, stem diameter, number of flowers, number of branches, and SLA (Figure S2, Supplementary Materials). The comparison between quadratic and linear models (not presented here) on the basis of the full dataset (*N* = 140) showed that the relationship between shade and trait values was better fitted by a quadratic function for all traits except for the number of branches (*p* < 0.05 for plant height, internode length, stem diameter, number of flowers, and SLA; see Figure S2, Supplementary Materials). The curvature of these quadratic reaction norms indicated that the effect of shade on traits changed sign beyond 45% shade. Around 70% shade, there was a “saturation” effect, and traits describing growth, development, and flowering collapsed. This level of shade, therefore, represents the threshold in these experimental conditions beyond which snapdragon plants are no longer responsive in a consistent way. In contrast, SLA increased exponentially as a result of both an increase in surface and a decrease in the dry mass of leaves (data not shown). Thereafter, we excluded the 70% shade treatment and considered only changes in traits that occurred between full light and 45% shade (*N* = 105). Up to 45% shade, the relationship with shade was significantly linear for most traits; plants grew taller, developed longer internodes, and featured more branches, while leaves were characterized by higher SLA. No such linear effects were detected for stem diameter and number of flowers (Figure 1; a bar diagram representation is shown in Figure S3, Supplementary Materials).

These results were confirmed when we included the identity of highly inbred lines as a random effect in a GLMM. We found a significant linear effect of shade (from 0 to 45%, *p* < 0.01) on the same four traits (plant height, internode length, number of branches, and SLA) and no significant effect of shade on stem diameter and number of flowers (Table S1, Supplementary Materials). Trait values measured in full light conditions significantly varied amongst lines, as illustrated by the significant line effect on the intercept of the reaction norm to shade for all traits except for plant height (internode length, stem diameter, number of flowers, number of branches, SLA, *p* < 0.001; see Table S1, Supplementary Materials). In contrast, the lines did not vary in how they reacted to shade, as illustrated by the lack of difference between the slopes of their reaction norms (i.e., the linear effects of shade on traits; see Table S1, Supplementary Materials). This lack of significance did not result from a lack of statistical power (Table S1, Supplementary Materials). It is interesting to note that lines explained 31% to 75% of the variation of traits, which was larger than the 5% to 43% of trait changes caused by shade (see corresponding *R*^2^ for shade effects and effects of line on the intercept in Table S1, Supplementary Materials).

### 3.2. Relationship between Shade and Global DNA Methylation

**Figure 2:**
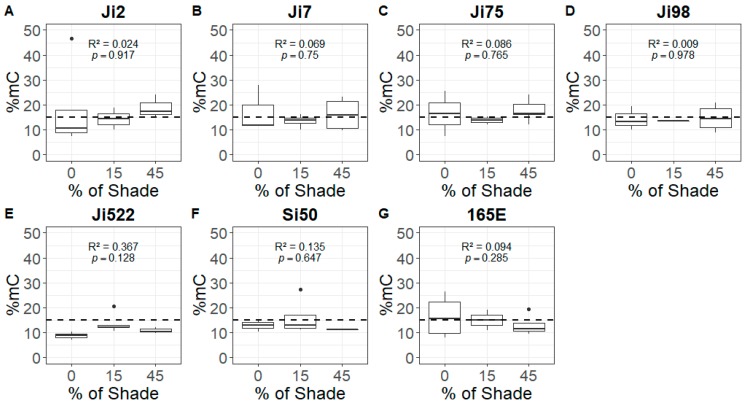
Global DNA methylation variation with shade in highly inbred lines of *A. majus.* Global DNA methylation percentages (%mC) as a function of shade treatments for every highly inbred line: Ji2 (**A**), Ji7 (**B**), Ji75 (**C**), Ji98 (**D**), Ji522 (**E**), Si50 (**F**), and 165E (**G**). The dashed line represents the mean %mC (14.92%). Coefficients of determinations (R^2^) and test probabilities (*p*) are given for each test of the relationship between the %mC and shade. *N* = 105 (five plants × three shade treatments × seven lines); each boxplot represents the mean and standard error for five plants.

The HPLC analysis, which was based on *N* = 84 successful extractions (three to five plants × three shade treatments × seven lines), revealed that about 15% of cytosines were methylated in the shoot apex of *A. majus* lines. We found no significant change in global DNA methylation percentage (%mC) in response to shade when we analyzed pooled data across lines (*p* = 0.87) and data of each line separately (Figure 2). These results were confirmed when we included the identity of lines as a random effect in a GLMM. We found no significant effect of shade (*R*^2^ = 0.00042, χ^2^ = 0.0301, *p* = 0.862) on %mC. Measurements of %mC in full light conditions did not vary amongst lines, as illustrated by the lack of line effect on the intercept (*R*^2^ = 0.0052, χ^2^ = 0, *p* = 1). Lines also did not vary in how their %mC varied with shade, as illustrated by the lack of difference between the slopes of %mC response to shade (*R*^2^ = 0.092, χ^2^ = 0, *p* = 1).

### 3.3. Relationship between Traits and Global DNA Methylation

We found no significant linear relationship between %mC and trait values (GLM, data not shown). These results were confirmed when we included the identity of lines as a random effect in a GLMM (Table S2, Supplementary Materials). Lines varied significantly in the position of the intercept of their relationship between %mC and trait values for four traits (internode length, stem diameter, number of flowers, and number of branches, *p* < 0.001). This means that trait values measured for %mC baseline levels varied significantly between lines in these four traits (Table S2, Supplementary Materials). The slope of the relationship between the %mC and trait values varied significantly between lines for the number of branches (*p* < 0.01; Table S2 and Figure S4, Supplementary Materials). The lack of significance for most traits did not result from lack of statistical power, but for the analysis of plant height which had low power (Table S2, Supplementary Materials). The significant result for the number of branches, therefore, was not revealed to be spurious.

### 3.4. Relationship between the Slopes of the Reaction Norms and the Global DNA Methylation Changes

**Figure 3:**
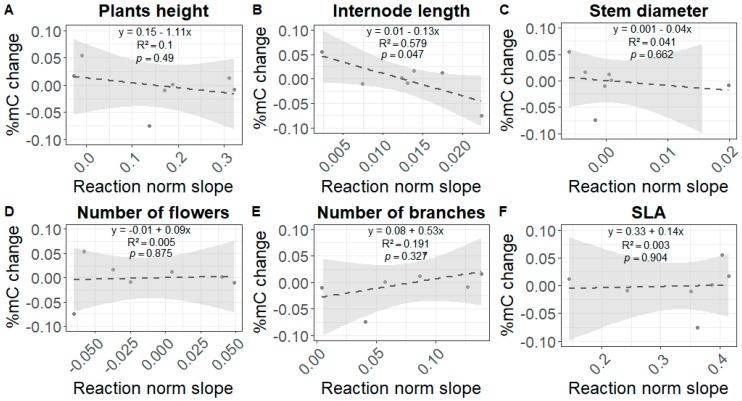
Relationship between the strength of shade avoidance responses and global DNA methylation changes. Changes in global DNA methylation percentages (%mC) as a function of the magnitude of the reaction norm to shade of plants. Both %mC change and reaction norm magnitude in response to shade are expressed in terms of slope coefficients for height in cm (**A**), internode length in cm (**B**), stem diameter in mm (**C**), number of flowers (**D**), number of branches (**E**), and SLA in m^2^∙kg^−1^ (**F**). Equations, test probabilities, and coefficients of determination (*R*^2^) are given for each trait. The gray area represents the 95% confidence interval around the dotted line, which represents the regression line. Each dot represents one highly inbred line. Statistical power of the chi^2^ tests for the effect of the %mC change on the slope of the reaction norm was estimated using power analyses defined by Cohen (pwr package in R): 13.3% for plant height, 99.8% for internodes length, 6.3% for stem diameter, 5% for the number of flowers, 35.9% for the number of branches, and 5% for the SLA.

In order to take into account the fact that only plants reacting to shade are expected to undergo changes in %mC, we compared the slope of the reaction norm to shade—the magnitude of phenotypic plasticity—to the slope of %mC modifications associated with shade for each trait. We found a negative relationship for internode length (*R*^2^ = 0.5792, *p* = 0.0469; Figure 3), which means that lines that were more plastic for this trait were also those that saw their percentage of global methylation most reduced. Low statistical power was recorded for the analysis of most traits except for internode length (Figure 3). The lack of significance found for most traits might be due to our inability to detect an effect when this effect is small. The relationship detected for internode length cannot be considered as spurious on the basis of power analyses. However, caution must nevertheless be taken in the interpretation of this *p*-value because it is close to the significance threshold.

## 4. Discussion

### 4.1. Phenotypic Shade Avoidance Response

Our results brought evidence for phenotypic plasticity in response to shade in highly inbred lines of *A. majus*. The reaction norm to shade was detected in two-thirds of the measured traits, demonstrating the existence of a shade avoidance syndrome in *A. majus*. The plastic response to shade of *A. majus* included plant height, internode length, number of branches, and SLA—traits that were already widely described as parts of the shade avoidance syndrome of other plants [26,27]. Although increases in plant height, internode length, and SLA are commonly reported in response to shade, increased branching is not. For instance, reduced branching is usually expected in response to shade as a result of apical dominance [26]. In contrast, increased branching was already described in myrtle (*Myrtus communis*), where shade led to more compact plants with more branches [75]. This might have reflected a strategy to increase light capture.

It is important to note that we did not characterize the shade avoidance response in natural populations. Our results were obtained in highly inbred lines of *A. majus* that are used for the genetic study of plant development and the selection of horticultural varieties. These lines underwent multiple generations of self-fertilization and cultivation in controlled conditions, which resulted in reduced levels genetic diversity. They also likely underwent genetic and phenotypic divergence from natural populations. Phenotypic responses to light changes were already described in *Antirrhinum* cultivars [48,49,76]. Collectively, these findings suggest that the SAR is widely conserved in *A. majus* artificially selected lines. One originality of our results resides in showing that the reaction norms of lines were comparable, although these lines were characterized by different phenotypes. This finding implies the existence of standing genetic variation for the traits involved in the shade avoidance response, as well as the absence of such variation for the type or magnitude of the shade avoidance response.

### 4.2. Global DNA Methylation

Our results showed that 15% of cytosines were methylated in the *A. majus* genome (~400 to 500 Mb). This is almost three times more than *A. thaliana* with ~5% for ~135 Mb, and the same order of magnitude as several other species, e.g., *Brassica oleracea* with ~16% for ~700 Mb, *Lepidium sativum* with ~15% for ~380 Mb, and *Primula vulgaris* with ~14% for ~500 Mb [60]. Our finding, therefore, corroborates that global DNA methylation (%mC) varies among plant species in relation to the genome size in angiosperms [60]. At the population level within species, epigenetic studies are challenging [77,78]. As discussed by Richards (2008) [78], the correspondence between the epigenetic and the genetic variations can reflect a wide spectrum of interrelations, ranging from their total dependence to their total independence. At the within-species level of *A. majus* lines, we found no differences in terms of %mC. Our results also showed that there was no variation of %mC with trait values. Our results, therefore, contrast with those from the literature that often report %mC variation within and between genotypes, and %mC variation associated with developmental processes [63,65,70,79,80,81]. Thus, two hypotheses could explain our results. The lack of %mC variation might be independent from the genetic variation between *A. majus* lines. Alternatively, %mC variation might be linked to genetic variation in *A. majus* lines, but their genetic differentiation might be too weak to detect %mC differences.

We found different results for the number of branches. Its association with %mC varied between lines (Figure S4, Supplementary Materials). This likely reflects genetic variation between lines for the number of branches. This result corroborates the usual finding that DNA methylation levels are often associated with genetic variation. An example in point is the quantitative genetic analysis of DNA methylation in natural populations of poplar submitted to water stress. In this example, the heritability of DNA methylation increased in the presence of the environmental constraint, which suggests that changes in DNA methylation were associated with genetic variation [63]. Several mechanisms can potentially underlie this type of link. For example, DNA methylation variability explained by DNA sequence variability might lie between 22% and 80% in humans because genetic polymorphism at a methylated cytosine site results in methylation variation [77]. Conversely, TEs, for which mobility across the genome is controlled by DNA methylation, can affect genetic variation [15]. Methylation is also known to affect DNA mutation rates [82] and, therefore, DNA polymorphism as a result of the spontaneous deamination of mC [83]. As underlined by our study, the diversity of epigenetic interactions with genetic variation limits our interpretation of how epigenetic mechanisms shape DNA methylation variation at the level of genetically diverse groups (e.g., populations and lines).

### 4.3. The Link between Phenotypic Plasticity and Epigenetic Changes

We found consistent levels of %mC across shade treatments when using models that did not account for potential genetic variation between *A. majus* lines. When several genotypes are investigated, this lack of direct association between shade and %mC might reflect that plants not reacting to shade are not expected to undergo %mC changes. When we took this information into account, our results showed that the increase in internode length in response to shade was negatively correlated to changes in %mC in response to shade. This supports the hypothesis that the magnitude of this reaction norm was associated with the global demethylation of the genome in response to shade. However, no such significant relationship was detected for the five other traits under study. Stem elongation in response to shade is commonly found in the shade avoidance response of plants [26,27]. This plastic response is often characterized by large modifications of the phenotype. Furthermore, its ecological significance in wild populations where it favors plants in competition for light is widely acknowledged [35,36,37]. Stem elongation in response to shade was already found to be associated with the global demethylation of the genome in *Stellaria* plants [46]. One might speculate that stem elongation is, therefore, the best candidate trait amongst the six traits under study to investigate the role of epigenetic variation in the shade avoidance response of plants. SLA should, however, not be discarded as a good candidate in *A. majus* based on our assessment. This is because (i) it is widely acknowledged as a highly plastic trait influenced by light in most plant species and was one of if not the most responsive to shade in our study, and (ii) we have to keep in mind that DNA methylation is tissue-specific [80,84]. Even if the meristem is where leaves are differentiated, tissue samples directly coming from leaves might be necessary to properly assess the association between the SLA response to shade and methylation changes.

Our finding of a correlation between plasticity and methylation changes does not imply causality. It simply opens up the question of this causality. It is widely acknowledged that gene expression can be controlled “directly” via methylation of gene promoters or bodies [16]. Gene expression can also be controlled “indirectly” through the activation of TE mobility, affecting promoters or acting as *cis* regulatory elements [15]. The mechanism linking demethylation at the level of the whole genome or multiple specific genes to the plastic response of internode growth remains to be investigated in *A. majus*. This will require assessing the control of gene expression and TE mobility via DNA methylation. Before going into such a thorough investigation, our finding will, however, need to be replicated. This is because our data must be interpreted with caution. The correlation between epigenetic changes and plasticity was found in only one trait and, although the size effect was large, and the relationship was statistically significant, the rejection probability was close to the threshold limit of non-significance (*p* = 0.0469). This test would not remain significant after a Bonferroni correction, but such a correction is also known to generate false negative results. The global DNA methylation percentage of a genome is also difficult to interpret because it provides us with an average value across the genome. As a consequence, new methylation and demethylation events occurring in similar proportions are masked by each other. The lack of %mC differences that we observed between lines and between shade treatments might hide changes in DNA methylation, resulting in the same amount of hypomethylated and hypermethylated genes and non-coding regions (TEs, repeated sequences). This level of detail might be revealed by epigenome sequencing (e.g., WGBS, EpiGBS, meDIP) at the DNA base or genomic region level [56,66]. With the publication in 2019 of a reference genome for *A. majus* [55], the feasibility of detailing the DNA methylation remodeling in response to shade in snapdragon will improve.

## 5. Conclusions

For the past few decades, the interest in epigenetic modifications and their role in the regulation of adaptive genes with ecological significance grew widely. Some studies provided evidence that DNA methylation has the potential to play a role in local adaptation and fast adaptive responses [85,86]. The relative contribution of genetic and epigenetic variation nevertheless remains to be assessed. Testing for the epigenetic basis of an ecologically significant phenotypic response to environmental stimuli is a first step toward the evaluation of the adaptive significance of DNA methylation [87]. Our findings in highly inbred lines of *A. majus* imply the existence in this species of a shade avoidance syndrome, which is an ecologically significant case of phenotypic plasticity widely found in plants. They also imply that the global genome demethylation of these lines was possibly associated with stem elongation in response to shade. Our study, therefore, supports the hypothesis that epigenetic variation might play a role in an ecologically significant phenotypic plastic response in plants. However, no such result was found for the other traits of the syndrome. Assessing this type of association by measuring global DNA methylation percentages can be done at the population level, at which ecological relevance can be tested. However, our study underlined some limits of this approach, and caution must be taken with the results that it generated. Further evidence at the DNA sequence level appears necessary before a solid conclusion can be drawn.

## Supplementary Materials

The following are available online at https://www.mdpi.com/2073-4425/10/4/256/s1: Figure S1: Average light spectrum and intensity analysis by spectrophotometer acquisition over 15 minutes of recording on a sunny day in one location for each treatment; Figure S2: Shade avoidance response in *A. majus* highly inbred lines; Figure S3: Average trait values across different levels of shade; Figure S4: Global DNA methylation variation with the number of branches in highly inbred lines of *A. majus*; Table S1: Effects of shade and highly inbred lines on phenotypic traits; Table S2: Effects of methylation and highly inbred lines on phenotypic traits.
