# Supplementary material for: Assessing Global DNA Methylation Changes Associated with Plasticity in Seven Highly Inbred Lines of Snapdragon Plants (Antirrhinum majus)
Source: Genes (Basel). 2019 Mar 28;10(4):256. doi: 10.3390/genes10040256 (PMC6523709; doi:10.3390/genes10040256)
Supplement: Supplementary file 1 [file genes-10-00256-s001.pdf]

Assessing global DNA demethylation during shade avoidance response of seven highly inbred lines of snapdragon plants (*Antirrhinum majus*)

Delphine GOURCILLEAU[1], Mathilde MOUSSET[1], Mathieu LATUTRIE[1], Sara MARIN[1], Alain DELAUNAY[2], Stéphane MAURY[2] and Benoît PUJOL[1,3]

[1.] Laboratoire Évolution & Diversité Biologique (EDB, UMR 5174), Université Fédérale de Toulouse Midi-Pyrénées, CNRS, IRD, UPS. 118 route de Narbonne, Bat 4R1, 31062 Toulouse cedex 9, France.

[2.] Laboratoire de Biologie des Ligneux et des Grandes Cultures (LBLGC, EA 1207 USC 1328 INRA). Université Orléans, 45067 Orléans, France.

[3.] PSL Université Paris: EPHE-UPVD-CNRS, USR 3278 CRIOBE, Université de Perpignan, 52 Avenue Paul Alduy, 66860 Perpignan Cedex, France.

* Correspondence: Corresponding authors: delphine.gourcilleau@gmail.com and benoit.pujol@univ-perp.fr

SUPPLEMENTARY FILES

Figure S1. Average light spectrum and intensity analysis by spectrophotometer acquisition over 15 minutes of recording on a sunny day in one location for each treatment. Colors represent the different levels of shading; yellow represents natural sunlight in the greenhouse and the three darker shades of gray represent 15%, 45%, and 70% shading effects, respectively.

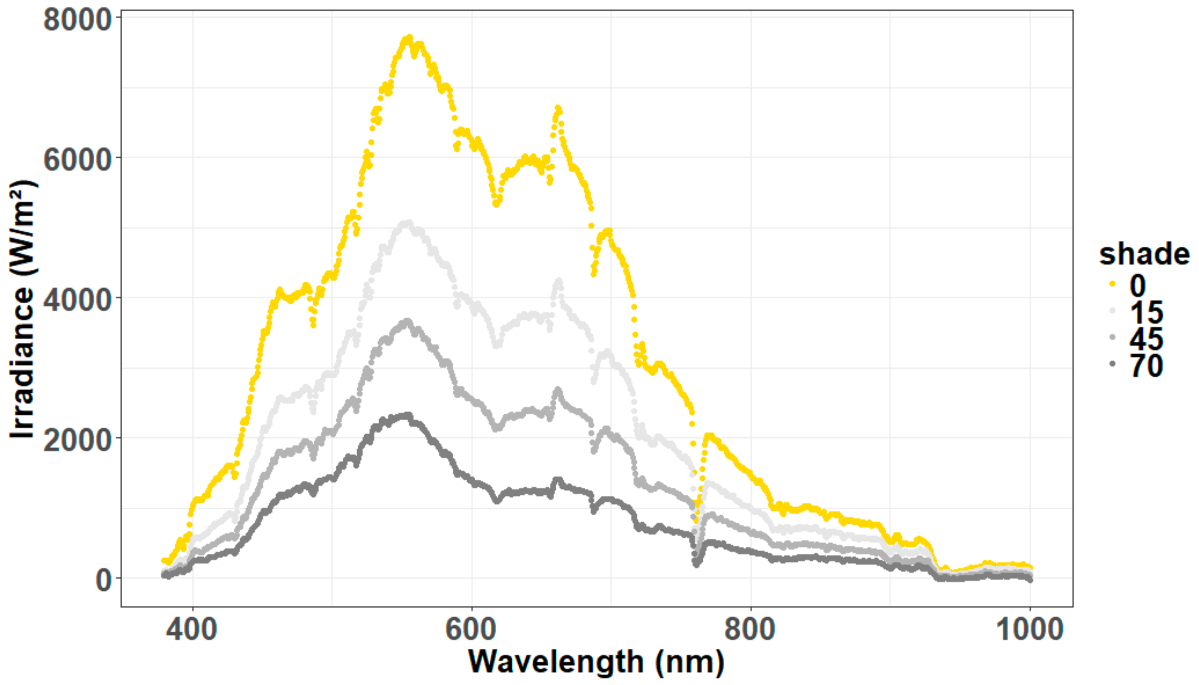

Figure S2. Shade avoidance response in *A. majus* highly inbred lines. Quadratic (A) and linear (B) relationships established by GLMs between phenotypic traits and shade. Phenotypic traits include plant height in cm, internode length in cm, stem diameter in mm, number of flowers, number of branches, and SLA in $m^2{\cdot}kg^{-1}$. Shade treatments include 0%, 15%, 45%, and 70%. Equations, probabilities of significance (p), coefficients of determination (R^2), and Akaike information criteria (AIC) are given for each relationship. $N = 140$ (five plants × four shade treatments × seven lines).

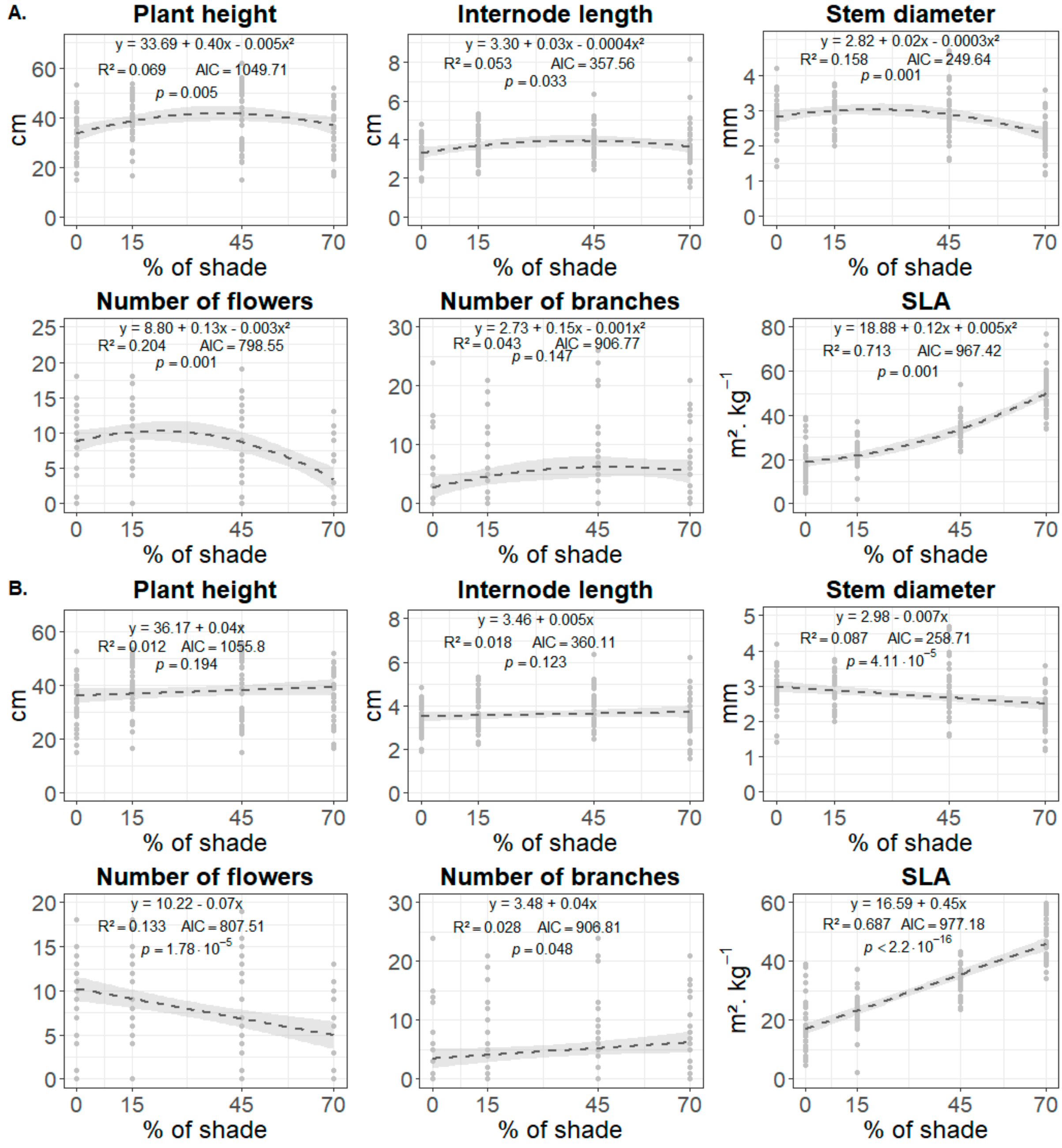

Figure S3. Average trait values across different levels of shade. Plant height in cm (A), internode length in cm (B), stem diameter in mm (C), number of flowers (D), number of branches (E), and SLA in $m^2 \cdot kg^{-1}$ (F) for different shade treatments (0%, 15%, and 45%). The seven lines are pooled. Coefficients of determinations (R^2) and test probabilities (p) are given for each test of the relationship between the trait values and shade. $N = 105$ (five plants × four shade treatments × seven lines); each boxplot represents 35 plants.

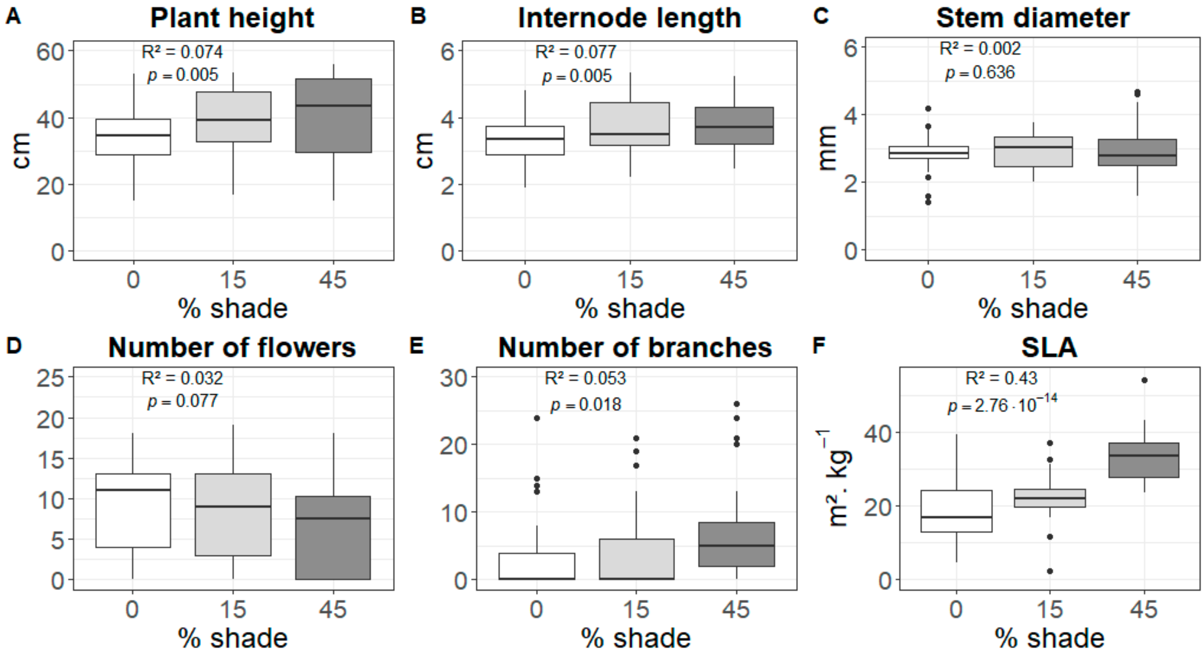

Figure S4. Global DNA methylation variation with the number of branches in highly inbred lines of *A. majus*. Global DNA methylation percentages (%mC) as a function of the number of branches for every highly inbred line: Ji2 (A), Ji7 (B), Ji75 (C), Ji98 (D), Ji522 (E), Si50 (F), and 165E (G). *N* = 15 (five plants × three shade treatments).

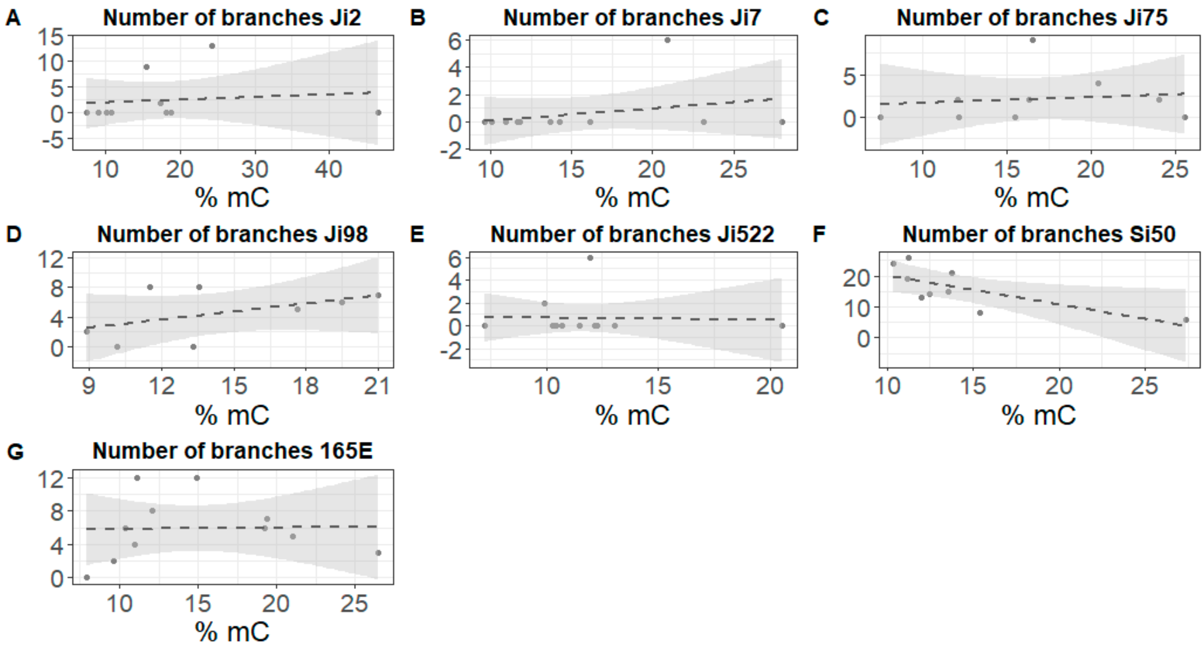

Table S1. Effects of shade and highly inbred lines on phenotypic traits. Coefficients of determination (R^2), chi-square values (χ^2), and test probabilities (p) for the effect of shade on traits, the effect of lines on the intercept of the trait reaction norm to shade, and the effect of lines on the slope of this reaction norm (generalized linear mixed model; GLMM). Correlations are reported significant as $p \leq 0.05$ (*), $p \leq 0.01$ (**), and $p \leq 0.001$ (***). $N = 105$ (five plants × three shade treatments × seven lines). Statistical power of the chi-square tests for the effect of line on slope was estimated using power analyses defined by Cohen (pwr package in R): 39.8% for plant height, 98% for internode length, 85.8% for stem diameter, 98.3% for the number of flowers, 100% for the number of branches, and 99.9% for the specific leaf area (SLA).

	**Plant height**			**Internode length**			**Stem diameter**			**Number of flowers**			**Number of branches**			**SLA**		
	R^2	χ^2	p	R^2	χ^2	p	R^2	χ^2	p	R^2	χ^2	p	R^2	χ^2	p	R^2	χ^2	p
**Shade**	**0.073**	**8.712**	**0.003** **	**0.076**	**11.42**	**0.001** ***	0.002	0.315	0.575	0.001	0.297	0.586	**0.052**	**18.45**	**1×10^{-5}** ***	**0.432**	**65.38**	**6×10^{-16}** ***
**Line on intercept**	0.163	2.789	0.095	**0.381**	**20.42**	**6×10^{-6}** ***	**0.311**	**20.42**	**6×10^{-6}** ***	**0.392**	**28.21**	**1×10^{-7}** ***	**0.753**	**104.8**	**2×10^{-16}** ***	**0.527**	**8.092**	**4×10^{-3}** **
**Line on slope**	0.166	0.198	0.906	0.392	1.143	0.565	0.296	0	1	0.399	0.694	0.707	0.757	1.592	0.451 **	0.505	0	1

Table S2. Effects of methylation and highly inbred lines on phenotypic traits. Coefficients of determination (R^2), chi-square values (χ^2), and test probabilities (p) for the effect of methylation (%mC) on traits, the effect of lines on the intercept of the relationship between %mC and trait values, and the effect of lines on the slope of this relationship. Correlations are reported significant as $p \leq 0.05$ (*), $p \leq 0.01$ (**), and $p \leq 0.001$ (***). $N = 105$ (five plants × three shade treatments × seven lines). Statistical power of the chi-square tests for the effect of line on slope was estimated using power analyses defined by Cohen (pwr package in R): 21.1% for plant height, 88.7% for internode length, 69.7% for stem diameter, 98.9% for the number of flowers, 99.9% for the number of branches, and 17.9% for the SLA.

	**Plant height**			**Internode length**			**Stem diameter**			**Number of flowers**			**Number of branches**			**SLA**		
	R^2	χ^2	p	R^2	χ^2	p	R^2	χ^2	p	R^2	χ^2	p	R^2	χ^2	p	R^2	χ^2	p
**%mC**	0.010	0.533	0.465	0.036	1.556	0.212	0.021	1.077	0.299	0.028	2.411	0.121	0.001	0.040	0.842	0.034	2.247	0.134
**Line on intercept**	0.093	1.020	0.312	**0.377**	**14.30**	**1×10^{-4}** ***	**0.293**	**9.703**	**1×10^{-3}** **	**0.464**	**20.20**	**6×10^{-6}** ***	**0.653**	**45.42**	**1×10^{-11}** ***	0.107	0.629	0.428
**Line on slope**	0.138	0.575	0.750	0.379	0.210	0.900	0.296	1.173	0.556	0.508	3.049	0.218	**0.718**	**12.16**	**0.002** **	0.124	0.645	0.724
